# A Genomic Sequencing Approach to Newborn Mass Screening and Its Opportunities

**DOI:** 10.1001/jamanetworkopen.2025.38198

**Published:** 2025-10-17

**Authors:** Diana Carli, Paola Quarello, Francesco Porta, Celeste Cagnazzo, Giulia Zucchetti, Camilla Francesca Proto, Rebecca Gianasso, Elisa Biamino, Caterina Carbonara, Alessandra Coscia, Caterina Parlato, Beatrice Fenoglio, Simonetta Guarrera, Marco Spada, Alessandro Mussa, Saverio Minucci, Franca Fagioli

**Affiliations:** 1 Department of Medical Sciences, University of Turin, Turin, Italy; 2 Immunogenetics and Transplant Biology Unit, Città della Salute e della Scienza di Torino, Turin, Italy; 3 Paediatric Onco-Hematology, Stem Cell Transplantation and Cellular Therapy Division, Regina Margherita Children’s Hospital, Turin, Italy; 4 Department of Paediatric and Public Health Sciences, University of Turin, Turin, Italy; 5 Paediatric Inborn Errors of Metabolism, Department of Paediatrics, Regina Margherita Children’s Hospital, Turin, Italy; 6 Department of Paediatrics, Regina Margherita Children’s Hospital, Turin, Italy; 7 Neonatal Intensive Care Unit, Sant’Anna Hospital, Città della Salute e della Scienza di Torino, Turin, Italy; 8 Neonatal Unit, Sant’Anna Hospital, Città della Salute e della Scienza di Torino, Turin, Italy; 9 Italian Institute for Genomic Medicine, Istituto di Ricovero e Cura a Carattere Scientifico, Candiolo, Italy; 10 Paediatric Clinical Genetics Unit, Regina Margherita Children’s Hospital, Turin, Italy

## Abstract

**Importance:**

Next-generation sequencing (NGS) has the potential to revolutionize newborn screening (NBS) by enabling the early identification of actionable genetic conditions not detectable by traditional methods. However, its integration into routine screening programs remains limited due to challenges related to feasibility, interpretation, and clinical impact.

**Objective:**

To investigate the acceptability and feasibility as well as the clinical implications of an NGS-based NBS program using DNA extracted from dried blood spots (DBSs).

**Design, Setting, and Participants:**

This prospective, nonpharmacological, interventional, single-center cohort study was proposed to parents whose newborns were delivered between October 1, 2023, and July 31, 2024, in a tertiary-level hospital in Turin, Italy, serving as a referral center for high-risk pregnancies. Newborns were consecutively selected at birth and enrolled after obtaining parental consent during the maternity stay.

**Exposures:**

Whole-exome sequencing (WES) using an in silico panel of 521 genes associated with pediatric-onset, actionable conditions. Only pathogenic or likely pathogenic variants were reported.

**Main Outcomes and Measures:**

Primary outcome was the technical feasibility of WES on DBSs. Secondary outcomes were establishment of a genomic sequencing database for rapid reanalysis if subsequent clinical indications arise and preliminary qualitative evaluation of selected example cases to explore the clinical implications of NGS-based NBS results to inform future implementation in routine care.

**Results:**

Of 4709 eligible newborns, 4067 (86.4%) were enrolled (2078 males [51.1%]; mean [SD] gestational age, 38.7 [1.9] weeks). Sequencing was successful in 4054 of newborns (99.7%). Of 4054 newborns analyzed, 542 (13.4%) screened positive. After orthogonal confirmation and family segregation studies, 568 actionable molecular diagnoses were retained, affecting 529 newborns (13.0%). During follow-up, 45 newborns underwent WES reanalysis due to clinical suspicion of a genetic disease, of whom 9 (20.0%) received a diagnosis not included in the initial screening panel.

**Conclusions and Relevance:**

Genomic NBS using WES from DBSs was technically feasible and allowed early identification of actionable genetic conditions. Integration of genomic methods into NBS, although ethical, logistical, and clinical challenges remain, is a logical step but requires adequate genetic counseling and long-term follow-up.

## Key Points

**Question:**

Can genomic sequencing on newborn dried blood spots enable early detection of actionable genetic conditions beyond traditional newborn screening?

**Findings:**

In this cohort study of 4054 newborns, 13.0% received at least 1 possible diagnosis based on pathogenic or likely pathogenic variants detected by a 521-gene whole exome sequencing–based panel. Genomic screening demonstrated feasibility, high acceptability, and potential clinical utility, with possible implications for early intervention, and identification of at-risk family members.

**Meaning:**

These results suggest that neonatal mass genomic sequencing is feasible and can complement traditional biochemical screening both by confirming and expanding the early detection capability of actionable genetic conditions.

## Teaser

This cohort study examines the feasibility, potential utility, and clinical implications of next-generation sequencing–based newborn screening.

## Introduction

Since its inception in the 1960s, mass newborn screening (NBS) has evolved substantially. Robert Guthrie’s discovery that phenylalanine could be detected on a dried blood spot (DBS) led to widespread adoption of NBS for phenylketonuria,^1^ marking a paradigm shift from treatment to prevention. A second revolution occurred in the 1990s with tandem mass spectrometry enabling simultaneous detection of multiple biomarkers.^2^ The Italian NBS program, one of the most extensive in Europe,^3^ screens for over 40 disorders. In the 2000s, next-generation sequencing (NGS) allowed parallel analysis of multiple genes,^4^ prompting pilot studies on its application in NBS^5,6,7,8,9,10,11,12,13,14,15,16^ (eTable 1 in Supplement 1). As NGS typically requires high-quality DNA, several studies used fresh blood, cord blood, or saliva,^1,2,3^ and only few assessed DBSs,^4^ leaving clinical applicability uncertain. While traditional NBS detects abnormal metabolites or hormones indicative of dysfunction at birth, NGS-based NBS can identify pathogenic or likely pathogenic (P/LP) variants before any physiological signs appear, potentially revealing conditions that may manifest later or never clinically emerge. This early detection represents a paradigm shift, enabling diagnosis of conditions lacking measurable biomarkers.

Despite 2 decades of NGS experience, genomic NBS application remains limited due to practical and interpretive challenges, ethical concerns, and limited therapies for many conditions.^7,15,16^ However, recent advances in drug repurposing and precision medicine are gradually expanding treatment options for genetic diseases and are prompting a reconsideration of the diagnostic approach.^17,18,19^ Moreover, early diagnosis of genetic diseases can improve care, prevent complications, guide family risk assessment, and reduce both unnecessary interventions and costs. Additionally, implementing genomic NBS could provide insights into the natural history and true phenotypic spectrum of genetic diseases, catalyzing research for developing therapies for disorders currently considered too rare to justify dedicated efforts.^20^

Based on these premises, we sought to investigate the acceptability and feasibility of an NGS-based NBS program using DNA extracted from DBSs in a large cohort of consecutive newborns. We also explored selected aspects of its potential clinical implications.

## Methods

### Study Design

We designed NeoGen, a prospective, nonpharmacological, interventional, single-center, nonprofit cohort study. The Comitato Etico Territoriale Interaziendale A.O.U. Città della Salute e della Scienza di Torino approved this study. Written informed consent was obtained from parents or legal representative of child participants. We followed the Strengthening the Reporting of Observational Studies in Epidemiology (STROBE) reporting guideline.^21^

This study was proposed to all parents whose newborns were delivered between October 1, 2023, and July 31, 2024, at the Sant’Anna Hospital in Turin, Italy, a regional tertiary-level referral center for high-risk pregnancies, and who had the capacity to provide parental or legal representative consent for study participation and data processing. The study was explained and consent was obtained by a physician during the hospital stay. Language and cultural support services were available.

Among the demographic characteristics collected, the geographic origins of participants were reported by parents and reviewed through the hospital electronic health record system; categories included African, Asian, European, South American, and multiple nationalities. This information was collected in this study because geographic origin may be relevant for the interpretation of genetic data.

The primary outcome was assessment of the feasibility of using a whole-exome sequencing (WES)–based panel on DBS-derived DNA for population-scale genomic screening. The secondary outcomes were establishment of a genomic sequencing database to support rapid NGS reanalysis in response to emerging clinical concerns during follow-up and preliminary qualitative evaluation of selected example cases to explore the clinical implications of neonatal genomic screening results to inform future integration in routine care. The example cases were selected from the cases with positive screen results, as they enabled a preliminary qualitative analysis of both the immediate benefits and the potential challenges experienced by the patient and/or their family members.

### Gene Panel Selection Process

The selection of genes for the NGS panel was carried out by a multidisciplinary board, which included laboratory and clinical geneticists, pediatric subspecialists experienced in rare diseases, and clinical psychologists. The final list comprised 521 genes associated with 517 conditions (eTable 2 in Supplement 1).

Selection criteria included (1) strong to moderate gene-disease association; (2) association with childhood-onset or childhood- to adult-onset condition; (3) availability of effective treatments or preventive strategies; and (4) clinical utility of early diagnosis. Genes associated exclusively with adult-onset conditions were excluded, even if listed among the actionable genes by the American College of Medical Genetics and Genomics (eg, heterozygous *BRCA1/2*). The panel targeted a broad spectrum of conditions, including inborn errors of metabolism (n = 131), endocrine disorders (n = 104), hearing loss (n = 83), immunodeficiencies (n = 67), hematological disorders (n = 44), cardiovascular disorders (n = 30), neuromuscular disorders (n = 23), gastrointestinal disorders (n = 15), craniosynostosis syndromes (n = 15), cancer predispositions (n = 12), renal disorders (n = 4), and cystic fibrosis (n = 1).

### Sample Processing

For each newborn, a DBS was collected on day 3 of life using an additional Guthrie card, separate from routine NBS, and air-dried for 24 hours. DNA was extracted from three 0.4-mm punches (using DNeasy Blood and Tissue Kit; Qiagen). DNA concentration was measured (using Qubit dsDNA High Sensitivity Assay; ThermoFisher). Samples lower than 3 ng/μL were re-extracted and concentrated (using Concentrator Plus; Eppendorf).

### Sequencing and Data Analysis

Library preparation and enrichment were performed (using Illumina DNA Prep with Exome 2.5 Enrichment kit; 37.5-Mb target size). Paired-end sequencing (2 × 150 bp) was carried out (using NovaSeq 6000 platform; Illumina). Reads were aligned to the GRCh37/hg19 reference genome. Mean target coverage was at approximately 120×, with 98.0% and 97.5% of the target covered at 10× or greater and 20× or greater, respectively. Variant annotation relied on public gene mutation databases (ClinVar, COSMIC, dbSNP, and LOVD), population frequency databases (ExAC and gnomAD), and in silico prediction tools (AlphaMissense, CADD, GERP, MutationTaster, PolyPhen-2, REVEL, SIFT, and SpliceAI).^22,23^ Literature was consulted as needed to support interpretation. WES data were securely stored and retained for 4 years after the study, with reanalysis available on clinical request.

### Variant Interpretation and Confirmation

Variants were interpreted following the American College of Medical Genetics and Genomics guidelines,^24,25^ with additional refinements to reduce false-positive results in asymptomatic newborns. Specifically, the criterion PM1 was excluded if PM5 was present; PP2 was excluded in the presence of PM1; PP3 was excluded if AlphaMissense predicted the variant to be benign; and P/LP variants based only on PM2+PP3 were accepted only if also predicted to be damaging by AlphaMissense. These refinements were applied only in the absence of published case reports or multiple ClinVar submissions supporting pathogenicity. P/LP variants were reported if they (1) were in the 521-gene panel; (2) occurred in an allelic state compatible with disease (ie, heterozygous for dominant; homozygous or potential compound heterozygous for recessive); or (3) had potential dominant effect, if from genes with both dominant and recessive inheritance. Heterozygous carrier status for autosomal recessive conditions was not reported, except for conditions where carriers may be at risk (eg, X-linked recessive disorders in females). Copy number variants (CNVs) were reported only if involving genes in the panel, classified as P/LP,^26^ and present in an allelic state compatible with the disease.

Orthogonal methods used for variant confirmation included Sanger sequencing for single-nucleotide variants and small indels, multiplex ligation–dependent probe amplification for single-exon CNVs, and array-CGH 60K (Agilent). Conventional karyotyping was also used for larger CNVs or chromosomal abnormalities.

### Return of Results

Newborns with screen-positive results were recalled for evaluation by a multidisciplinary team, which included a psychologist, a pediatric geneticist, and condition-specific pediatric specialists. Clinical assessment and second-level investigations were conducted as needed to assess phenotype. Blood samples from the newborn and parents were collected for confirmation and familial segregation. Results were communicated during posttest genetic counseling, and follow-up was planned for the proband and, when appropriate, family members.

### Statistical Analysis

Summary statistics (means [SDs] and medians [ranges]) were used for continuous variables, and categorical data were presented as numbers and percentages. Statistical analyses were performed using IBM SPSS Statistics, version 29.0.2.0 (IBM Corp).

## Results

Of the 4709 eligible newborns, 4067 (86.4%) were enrolled (2078 males [51.1%], 1989 females [48.9%]; mean [SD] gestational age, 38.7 [1.9] weeks). Geographic origins of participants were as follows: 293 (7.2%) African, 89 (2.2%) Asian, 3532 (86.8%) European, 56 (1.4%) South American, and 97 (2.4%) multiple nationalities. Among the 642 newborns whose parents declined participation, the main reasons were linguistic barriers and general distrust of clinical research. Less frequent reasons included lack of interest and religious concerns. Demographic and perinatal information of the NeoGen population is summarized in Table 1.

**Table 1.** Demographic and Perinatal Characteristics of the NeoGen Population

Characteristic	Enrolled newborns, No. (%) (n = 4067)
Sex	
Male	2078 (51.1)
Female	1989 (48.9)
Geographic origin	
African	293 (7.2)
Asian	89 (2.2)
European	3532 (86.8)
South American	56 (1.4)
Multiple nationalities	97 (2.4)
Conception method	
Physiological	3686 (90.6)
Medically assisted	381 (9.4)
Birth weight, g	
<2500	416 (10.2)
2500-4000	3474 (85.4)
>4000	177 (4.4)
Gestational age, wk	
<37	371 (9.1)
37-40	2699 (66.4)
>40	997 (24.5)
Condition at birth	
Healthy	3730 (91.7)
NICU stay	337 (8.3)

Abbreviation: NICU, neonatal intensive care unit.

### Sequencing 

Sequencing was successful for 4054 of 4067 newborns (99.7%). DNA extraction failed in 53 cases, with 13 families declining additional sampling and 40 families consenting and being successfully sequenced. Sequencing failed in 19 participants, all of whom underwent resampling and were successfully analyzed. The overall screening failure rate was 0.3% (13 of 4067). The median (range) turnaround time from consent to report delivery was 32 (15-97) days. Recruitment and sequencing outcomes are summarized in the Figure.

**Figure.**
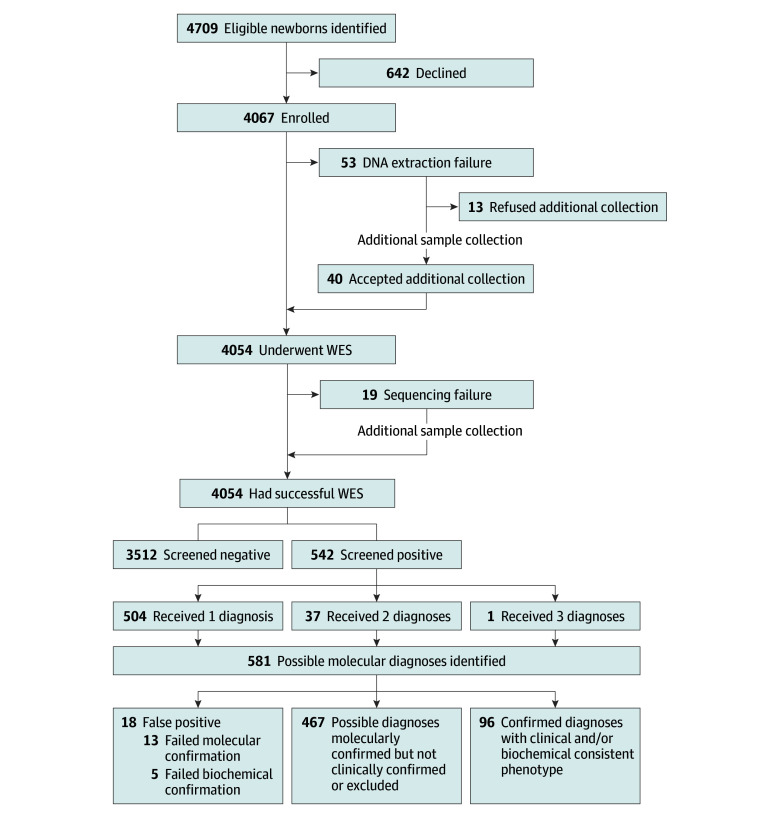
Flowchart of Newborn Enrollment and Whole Exome Sequencing (WES) Workflow

### Cases With Positive Screen Results

Overall, 542 of 4054 newborns (13.4%) had positive screen results. These cases included 481 of the 3717 (12.9%) in the well-baby nursery and 61 of the 337 (18.1%) in the neonatal intensive care unit (NICU). In total, 372 P/LP variants were identified in 132 genes or chromosomal regions, resulting in 581 possible molecular diagnoses: 504 newborns had 1 diagnosis, 37 newborns had 2 diagnoses, and 1 newborn had 3 diagnoses (Figure). Diagnoses were grouped into macroareas based on the primary phenotype (Table 2). The macroareas were cardiology (n = 179), hematology/immunology (n = 147), endocrinology (n = 92), and other (n = 163).

**Table 2.** Distribution of the 581 Possible Molecular Diagnoses Identified in 542 Newborns

Macroarea and subcategory	No. of diagnoses
Cardiology (n = 179)	
Cardiomyopathies	79
Arrhythmias	70
Aneurysms	30
Hematology/immunology (n = 147)	
G-6-PD deficiency	123
Coagulation disorders	18
Hemoglobinopathies	2
Other hematologic/immunologic disorders	4
Endocrinology (n = 92)	
Puberty disorders	42
Cholesterol metabolism disorders	19
Skeletal and bone metabolism disorders	9
Diabetes	9
Obesity	6
CAH (*CYP21A2* variant)	4
Thyroid and parathyroid disorders	3
Other (n = 163)	
Hearing loss	62
Metabolic disorders	31
Large CNVs	26
Craniosynostosis	13
Neuromuscular disorders	10
Cancer predisposition syndromes	10
GI disorders	6
Cystic fibrosis (*CFTR* variant)	5

Abbreviations: CAH, congenital adrenal hyperplasia; CNV, copy number variant; G-6-PD, glucose-6-phosphate dehydrogenase; GI, gastrointestinal.

Orthogonal confirmation was considered unnecessary in 126 of 581 diagnoses (21.7%), including 123 diagnoses of glucose-6-phosphate dehydrogenase deficiency and 3 diagnoses of biotinidase deficiency, as biochemical testing was deemed sufficient. The remaining 455 diagnoses (78.3%) were considered worthy of orthogonal confirmation, which was performed in 395 newborns. Parental segregation analysis was conducted in 206 families (eTable 3 in Supplement 1).

Overall, 13 of 581 diagnoses (2.2%) were classified as false-positive due to technical limitations. Seven of these false-positive diagnoses were not confirmed: 5 involved single-nucleotide variants or CNVs of low sequencing quality not validated by Sanger sequencing or multiplex ligation–dependent probe amplification, while 2 large CNVs potentially involving critical regions associated with microdeletion or microduplication syndromes were confirmed by array-CGH but did not actually involve the critical regions. An additional 6 diagnoses were considered false-positive following segregation analysis, which revealed 2 variants in cis in autosomal recessive genes. After their exclusion, 568 potential diagnoses remained across 529 newborns (13.0% of 4054).

Of the 568 retained diagnoses, 96 (16.9%) were clinically and/or biochemically confirmed in the newborn and/or in an affected parent and can therefore be considered true-positive diagnoses. Five diagnoses (0.9%) were excluded based on highly reliable biochemical testing and were also considered false-positive, bringing the total number of false-positive diagnoses to 18 of 581 (3.1%). The remaining 467 diagnoses (82.2%) are currently classified as possible diagnoses because they involve conditions with variable, incomplete, or age- and sex-dependent penetrance, which cannot be excluded based on a single normal clinical assessment. Notably, in 31 of these latter cases, the family history was suggestive of the suspected condition. Details on each newborn, diagnosis, and segregation analysis are provided in eTable 3 in Supplement 1. Twenty-seven variants were recurrently identified in 3 or more unrelated individuals (eTable 4 in Supplement 1).

### Comparison With Biochemical NBS

NGS-based NBS results were compared with those from biochemical NBS. Genes corresponding to conditions included in the biochemical NBS are indicated in eTable 2 in Supplement 1. Concordant biochemical and NGS-based NBS findings were observed in 2 newborns—1 with primary carnitine deficiency (*SLC22A5* gene) and the other with CblC deficiency (*MMACHC* gene). With NGS-based NBS, 3 newborns tested positive for potential partial biotinidase deficiency. With biochemical NBS, all 3 patients showed negative results and normal biotinidase activity at the retesting performed at the clinical recall, thereby excluding the diagnosis.

### Example Clinical Cases

Following are case examples to illustrate different clinical scenarios emerging from genomic screening. These cases do not represent the overall distribution of results.

#### Dual Diagnosis of Gaucher Disease and Usher Syndrome

NGS-based NBS identified biallelic P/LP variants in the *GBA* gene, and β-glucocerebrosidase deficiency was confirmed by enzymatic testing, consistent with Gaucher disease. The newborn is currently asymptomatic; enzyme replacement therapy will be initiated on symptom onset, according to current clinical guidelines. Additionally, biallelic P/LP variants were detected in the *USH2A* gene. Audiological evaluation at birth was normal, and the patient is under monitoring for potential late-onset Usher syndrome.

#### Long QT Syndrome

A newborn had a prenatal diagnosis of second-degree atrioventricular block and presented at birth with wide-complex tachycardia with torsade de pointes. NGS-based NBS revealed a heterozygous de novo P/LP variant in the *KCNH2* gene, confirming the diagnosis of long QT syndrome. The patient experienced recurrent arrhythmias and QT prolongation, requiring antiarrhythmic therapy, epicardial pacemaker implantation, and left thoracic sympathectomy.

#### Familial Cardiomyopathies

A newborn carried a paternally inherited P/LP variant in the *PKP2* gene. While the newborn was asymptomatic, the father had arrhythmogenic right ventricular cardiomyopathy and was unaware of the genetic basis of his condition. Similarly, another newborn carried a paternally inherited P/LP variant in the *DSP* gene. The patient was asymptomatic, and the father was later found to have an undiagnosed hypokinetic cardiomyopathy.

#### Dual Diagnosis of Neurofibromatosis Type 1 and Congenital Myasthenic Syndrome

A newborn had a prenatal diagnosis of a maternally inherited P/LP variant in the *NF1* gene, consistent with a family history of neurofibromatosis type 1. After birth, NGS-based NBS identified an additional maternally inherited P/LP variant in the *SYT2* gene associated with congenital myasthenic syndrome. The mother had mild neuromuscular symptoms but no prior diagnosis. The newborn, delivered preterm at 28 weeks and 4 days of gestation with severe intrauterine growth restriction, required noninvasive ventilation and enteral nutrition. Targeted treatment with 3,4-diaminopyridine was initiated with clinical benefit.

#### Mild Fibrillinopathy

A newborn carried a maternally inherited P/LP variant in the *FBN1* gene. While the newborn was asymptomatic, the mother presented subtle features suggestive of fibrillinopathy, including mitral valve prolapse, sternal asymmetry, mild dysmorphisms, and a family history of aortic aneurysm.

### WES Analysis in Symptomatic Newborns

During follow-up, 45 enrolled newborns underwent clinical evaluation for suspected genetic disorders, regardless of their NGS screening results. Evaluations occurred from birth to several months of age. Previously generated WES data were reanalyzed as singleton analyses, with parental segregation performed when indicated using methods such as Sanger sequencing. A molecular diagnosis was made in 9 cases (20.0%) involving conditions not covered by the NeoGen panel (Table 3).

**Table 3.** Confirmed and Newly Identified Genetic Diagnoses in Newborns Referred for Genetic Evaluation During Follow-Up

Initial screening result	Reason for genetic referral	Findings from WES reanalysis	Diagnosis
Negative	Mother carrier of X-linked ocular albinism	Hem *GPR143* (NM_000273.3) c.685G>C, p.Gly229Arg	Ocular albinism (OMIM 300500)
Negative	Skeletal dysplasia with disproportionate short stature, mild pulmonary stenosis, low-set posteriorly rotated ears	Het *ADAMTSL2* (NM_014694.4) c.1558_1568del, p.Asp520fs and c.715G>A, p.Ala239Thr	Geleophysic dysplasia (OMIM 231050)
Negative	Complex brain malformation	Het *TUBA1A* (NM_006009.4) c.481T>G, p.Tyr161Asp	Lissencephaly (OMIM 611603)
Negative	Macrocephaly and hypotonia	Het *CHD3* (NM_001005273.3) c.3682C>T, p.Arg1228Trp	Snijders Blok-Campeau syndrome (OMIM 618205)
Negative	Pierre Robin sequence	Het *COL2A1* (NM_001844.5) c.2403dup, p.Glu802Arg*22	Stickler syndrome (OMIM 108300)
Negative	Small for gestational age, hypotonia, dystonia	Het *GABRG2* (NM_198904.4) c.316G>A (p.Ala106Thr)	Developmental and epileptic encephalopathy (OMIM 618482)
Negative	Complex brain malformation	Het *TUBA1A* (NM_006009.4) c.352G>A, p.Val118Met	Lissencephaly (OMIM 611603)
Negative	Family history of cavernomas; known familial *KRIT1* variant	Het *KRIT1* (NM_194454.3) c.712C>T, p.Leu238Phe	Cerebral cavernous malformations (OMIM 116860)
Negative	Macrosomia and cardiac hypertrophy	Het *RIT1* (NM_006912.6) c.265T>C, p.Tyr89His	Noonan syndrome (OMIM 615355)

Abbreviations: Hem, hemizygous; Het, heterozygous; WES, whole exome sequencing.

## Discussion

In NeoGen, we found that large-scale neonatal sequencing on DBSs is feasible in a single-center setting. Overall, 13.0% of newborns had possible diagnoses included among the screened conditions. Yield comparisons with prior studies are limited by heterogeneity in study populations (eg, healthy vs NICU stay) and reporting strategies (eg, age of onset of reported disease; classification of reported variant)^5,6,7,8,9,10,11,12,13,14,15,16^ (eTable 1 in Supplement 1).

In our study, the molecular findings were relevant to clinical outcomes for several reasons. In some cases, NGS-based NBS confirmed diagnoses already suspected at biochemical NBS, demonstrating the value of this screening in providing rapid molecular confirmation. In other cases, it enabled the presymptomatic diagnosis and early management of conditions not included in traditional NBS. In this context, glucose-6-phosphate dehydrogenase deficiency was the most frequent potential diagnosis. Since hemolytic crises occur in approximately 20% of heterozygous females,^27^ the identification of carriers is also clinically relevant for both personal risk management and the detection of at-risk male relatives. Furthermore, NGS screening also enabled rapid molecular confirmation of clinically evident conditions not detected by traditional NBS. In some cases, findings in newborns uncovered previously unrecognized conditions in family members, allowing the identification and follow-up of affected relatives. In other cases, NGS-based NBS enabled early therapy initiation and/or the identification of dual diagnoses, avoiding diagnostic delays and missed conditions.

While the clinical utility of genomic screening was evident in these instances, its benefit was more controversial in others. For example, some P/LP variants in newborns or relatives were not associated with any clinical manifestations during follow-up. In such cases, the value of cascade testing was uncertain, as lack of supporting clinical signs may lead to psychological distress, overmedicalization, and strain on health care systems.^20^ For conditions with incomplete penetrance, a definitive diagnosis cannot be excluded, warranting long-term monitoring. In contrast, for conditions with complete early penetrance, normal confirmatory tests can reliably exclude the diagnosis,^28^ avoiding unnecessary cascade testing and follow-up. In other situations, P/LP variants were associated with mild presentations, raising uncertainty about diagnosis confirmation and management. For many genetic diseases, the full phenotypic spectrum remains poorly defined, and no standardized guidelines exist for follow-up in patients with no or subtle symptoms.^29^ In our cohort, although the molecular false-positive diagnosis rate was low, only 16.9% of diagnoses were clinically or biochemically confirmed and 0.9% were excluded. The remainder are currently unclassifiable and require longitudinal follow-up.

Another strength of NGS screening is the utility of stored sequencing data for subsequent reanalysis. In our study, WES analysis led to a diagnosis in 20.0% of newborns referred for genetic counseling due to suspected disorders. Early genomic sequencing may thus offer long-term benefits, enabling a new approach to diagnosing childhood-onset genetic conditions. Readily available data allow for rapid reanalysis, potentially within hours, reducing time to diagnosis and costs.^20^

In addition to its diagnostic value, this study confirmed the acceptability of NGS-based NBS in a clinical setting. The enrollment rate (86.4%) aligned with rates in prior pilot studies (36%-90%)^5,6,7,8,9,10,11,12,13,14,15,16^ (eTable 1 in Supplement 1), reflecting the growing public awareness and acceptance of genomics for early detection of rare disease. Turnaround times were comparable to times in other studies but still longer than for biochemical NBS, which typically yields results within 10 days.^30^ For patients in the NICU, the 30-day time frame is not aligned with current standards of rapid genomic testing, which, at our institution (as in many centers worldwide), is currently available only for selected cases and exclusively within a diagnostic, rather than a screening, framework. Although further process optimization may shorten turnaround times, infrastructures matching the speed of biochemical methods remain limited. This gap highlights the need for an integrated approach, combining biochemical and genomic screening rather than replacing one with the other. Moreover, biochemical NBS can detect diseases with abnormal analytes but no known genetic cause, such as certain types of congenital hypothyroidism. For these reasons, NGS-based NBS is unlikely to become a stand-alone first-tier test.^11,20,31^

### Limitations

This study has several limitations. First, although the selected 521-gene panel was extensive, relevant diagnoses may have been missed due to excluded genes. The use of the GRCh37/hg19 reference genome may also have limited variant detection and annotation in certain regions. Moreover, targeted WES has intrinsic limitations, such as poor breakpoint resolution and low sensitivity for large or complex structural variants, leading to possible false-positive results requiring orthogonal confirmation. Second, variant interpretation remains challenging: despite stringent criteria, false-positive results and uncertain findings may still arise, complicating clinical decisions and potentially impacting families psychologically. Third, long-term outcomes are lacking, especially for conditions with variable expressivity or incomplete penetrance, limiting the current ability to assess the appropriateness of including specific genes in NBS. Finally, NeoGen was a single-center study conducted in a tertiary care setting, and most enrolled newborns were of European origin, which may limit the generalizability of variant interpretation and allele frequency estimates to other ancestral groups.

## Conclusions

This cohort study demonstrated the feasibility and potential utility of genomic NBS using WES from DBSs. NBS is a major public health success, enabling early detection of treatable conditions. Genomic approaches broaden this potential by identifying more diseases but also introduce challenges such as variant interpretation, false-positive results, overmedicalization, and uncertain management of rare or mild conditions. Integrating NGS into NBS is a logical step but requires adequate genetic counseling and long-term follow-up. Pilot studies such as NeoGen are essential to address clinical, logistical, and ethical issues before broader adoption.

## Notes

Supplement 1. **eTable 1.** Summary of Previous Newborn Molecular Screening Projects **eTable 2.** Genes and Associated Conditions Included in the Neogen Panel **eTable 3.** Summary of Clinical and Genetic Findings in Screen-Positive Newborns **eTable 4.** List of Recurrent Genetic Variants Identified in ≥3 Unrelated Individuals

Supplement 2.
Data Sharing Statement
