# Supplementary material for: A Genomic Sequencing Approach to Newborn Mass Screening and Its Opportunities
Source: JAMA Netw Open. 2025 Oct 17;8(10):e2538198. doi: 10.1001/jamanetworkopen.2025.38198 (PMC12534850; doi:10.1001/jamanetworkopen.2025.38198)
Supplement: Supplement 2. — Data Sharing Statement [file jamanetwopen-e2538198-s002.pdf]

Data Sharing Statement

Carli. A Genomic Sequencing Approach to Newborn Mass Screening and Its Opportunities. *JAMA Netw Open*. Published October 17, 2025. doi:10.1001/jamanetworkopen.2025.38198

Data

Data available: All data are included in this article and its supplementary material; additional information is available from the corresponding author upon reasonable request.
